# Real-Time Reverse Transcription PCR Assay for Detection of Senecavirus A in Swine Vesicular Diagnostic Specimens

**DOI:** 10.1371/journal.pone.0146211

**Published:** 2016-01-12

**Authors:** Alexa J. Bracht, Emily S. O’Hearn, Andrew W. Fabian, Roger W. Barrette, Abu Sayed

**Affiliations:** 1 United States Department of Agriculture, Animal and Plant Health Inspection Service, Foreign Animal Disease Diagnostic Laboratory, Plum Island Animal Disease Center, Orient, NY, United States of America; 2 United States Department of Agriculture, Animal and Plant Health Inspection Service, Agriculture Select Agent Services, National Import Export Services, Riverdale, MD, United States of America; Auburn University, UNITED STATES

## Abstract

Senecavirus A (SV-A), formerly, Seneca Valley virus (SVV), has been detected in swine with vesicular lesions and is thought to be associated with swine idiopathic vesicular disease (SIVD), a vesicular disease syndrome that lacks a defined causative agent. The clinical presentation of SIVD resembles that of other more contagious and economically devastating vesicular diseases, such as foot-and-mouth disease (FMD), swine vesicular disease (SVD), and vesicular stomatitis (VS), that typically require immediate rule out diagnostics to lift restrictions on animal quarantine, movement, and trade. This study presents the development of a sensitive, SYBR Green RT-qPCR assay suitable for detection of SV-A in diagnostic swine specimens. After testing 50 pigs with clinical signs consistent with vesicular disease, 44 (88%) were found to be positive for SV-A by RT-qPCR as compared to none from a negative cohort of 35 animals without vesicular disease, indicating that the assay is able to successfully detect the virus in an endemic population. SV-A RNA was also detectable at a low level in sera from a subset of pigs that presented with (18%) or without (6%) vesicular signs. In 2015, there has been an increase in the occurrence of SV-A in the US, and over 200 specimens submitted to our laboratory for vesicular investigation have tested positive for the virus using this method. SV-A RNA was detectable in all common types of vesicular specimens including swabs and tissue from hoof lesions, oral and snout epithelium, oral swabs, scabs, and internal organ tissues such as liver and lymph node. Genome sequencing analysis from recent virus isolates was performed to confirm target amplicon specificity and was aligned to previous isolates.

## Introduction

Since the coincidental isolation of Seneca Valley virus (SVV), recently termed Senecavirus A (SV-A) [1], as a cell culture media contaminant in 2002, a number of serologically similar viruses were identified and grouped to the classification of *Senecavirus* [2]. The primary sequence analysis of the conserved polypeptide regions (P1, 2C, 3C and 3D) of the first isolate (SVV-001) showed that the virus is most closely related to cardioviruses in the family of *Picornaviridae* [2]. The single-stranded RNA genome of SV-A displays the secondary structural features of an internal ribosome entry site (IRES) that resembles the IRES element of classical swine fever virus (CSFV) of the family *Flaviviridae*, giving rise to the possibility that genetic exchange may have occurred between members of *Picornaviridae* and *Flaviviridae* during persistent co-infection in pigs [3]. Importantly, SV-A is a natural oncolytic agent, with the ability to selectively replicate in; and kill human tumor cells of neuroendocrine origin, thus, the virus is being advanced as a tool for potential therapeutic intervention of cancer [4].

Swine are considered to be the natural hosts of SV-A and all known SV-A sequenced isolates have been obtained from pigs. Previously, by regression analysis of partial genome sequences, it was suggested that different isolates of SV-A had a common ancestor and were assumed to have been introduced into the US pig populations (http://www.europic.org.uk/Europic2006/posters/Knowles.svv.01.pdf). Virus isolated in cell culture from tissue specimens of a diseased pig presenting vesicular lesions on the snout and feet in 2005, was identified by the National Veterinary Services Laboratories’ (NVSL) Foreign Animal Disease Diagnostic Laboratory (FADDL) as SV-A using a broad pan-viral microarray (unpublished data). More recently, this vesicular disease syndrome, with as yet unidentified etiology, has been termed swine idiopathic vesicular disease (SIVD) [5, 6]. Despite the isolation of SV-A in cell culture, FADDL has been unsuccessful at reproducing clinical signs by experimental inoculation of pigs with live virus. Negative observations were also made by other laboratories who conducted animal inoculations with multiple SV-A isolates [7]. Singh et al (2012) proposed SV-A as the causative agent of SIVD from a detailed clinical, diagnostic and histopathological study on a Chester White boar suffering from anorexia, lethargy, lameness and vesicular lesions [8]. However, association of SV-A with SIVD, or as the sole causative agent, is speculative at this time since the virus has also been isolated from pigs lacking clinical disease [2]. SIVD has been reported in pigs in the continents of North America and Australia [6, 9–11]. Although SIVD itself does not pose an economic concern, veterinary diagnosis from clinical signs is complicated since similar vesicular lesions can be formed due to common viral infections such as parvovirus, enterovirus, toxins in food supply, or burns [12–16]. Additionally, SIVD clinically resembles high consequence transboundary animal diseases (TADs) such as foot and mouth disease (FMD), swine vesicular disease (SVD), vesicular stomatitis (VS), and vesicular exanthema of swine (VES). A few laboratory methods have been developed for detection of SV-A including a virus serum antibody neutralizing test and a competitive enzyme linked immunosorbant assay (cELISA), which are not widely available [7, 17]. The principal aim of this study was to develop a specific real-time RT-PCR (RT-qPCR) assay for fast, sensitive, and quantitative detection of SV-A RNA in vesicular diagnostic tissues.

## Methods and Materials

### Diagnostic Specimens

Current and archived field specimens previously submitted to FADDL for routine diagnostic evaluation were used in this study and were diagnosed free of FMD, SVD, VES, and VSV. Clinical specimens naturally exposed to disease were collected by state or federal veterinarians from privately owned agricultural animals raised for food production and sent to our laboratory for diagnosis. These samples were provided for diagnostic purposes, and not specifically for this experiment. Additionally, no animals were used or experimentally infected to generate these field specimens for the purpose of this study, as such; no IACUC protocols apply or are available. Serum collections from non-clinical animals were made available from an ongoing surveillance program conducted by the Animal and Plant Health Inspection Service (APHIS) at FADDL.

### Virus Isolation

Positive control virus was isolated from swine vesicular lesion tissue obtained in 2007. A 10% homogenate (w/v) was made in Minimum Essential Medium Eagle containing 4% fetal bovine serum (Lonza) in a Retsch Mixer Mill 400 using a 25 ml stainless steel grinding jar and a single 20mm stainless steel grinding ball (Retsch). The homogenate was clarified by low speed centrifugation and passed through a sterile 0.45 micron filter (GE Healthcare). The filtered homogenate was used to inoculate a confluent monolayer of swine kidney (SK-6) and or Instituto Biologico Rim Suino-2 (IBRS-2) cell cultures [18–19]. Once cytopathic effects (CPE) were observed, the cell lysate was clarified by low speed centrifugation, and the supernatant was reserved as virus stock.

### Conventional RT-PCR Amplification

Total RNA from various specimens were performed using Magmax Total RNA Isolation kit (Life Technologies). The amplification of a 983 bp fragment from the polyprotein gene in the VP1 region was adopted from the conventional RT-PCR as previously described [http://www.europic.org.uk/Europic2006/posters/Knowles.svv.01.pdf]. Forward (SVV-1C556F) and reverse (SVV-2A22R) primers were added at concentrations of 40 pmol and 80 pmol respectively, in a mixture of Superscript III One Step RT-PCR w/ Platinum Taq HiFi (Invitrogen) for reverse transcription, and cycling amplification of: 30 min at 42°C, 2 min at 94°C, followed by 40 cycles of 15 sec at 94°C, 30 sec at 55°C and 1 min at 72°C.

### Positive Amplification Control

The 983 bp RT-PCR product derived from CPE-positive cell culture was purified using the QIAquick PCR Purification kit (Qiagen) and cloned into the plasmid vector pCR4-TOPO (Life Technologies) according to manufacturer’s protocols. DNA sequences of the plasmid inserts were confirmed using an ABI 3900 automated sequencer (Life Technologies). Sequences were assembled using Sequencher program (Gene Codes Corporation) and confirmed to be SV-A using the BLAST function of the National Center for Biotechnology Information (NCBI).

### Real-Time RT-PCR Amplification

Since the majority of SV-A sequences available in Genbank were derived from the VP1 coding region, two highly conserved short sequences, SV-A-qF (5`-GGGTAACACTGACACCGATTT) and SV-A-qR (5`-TCGAGATCGATCAAACAGGAAC) were selected to prime amplification of an 87 bp region located within the 983 bp conventional RT-PCR target sequence. RT-qPCR was performed using Power SYBR Green RNA-to-Ct 1-step kit (Life Technologies) according to the following thermal cycling conditions: 48°C for 5 min, 95°C for 10 min, 40 cycles of: 95°C for 5 sec and 60°C for 1 min, followed by a default stage of melting curve analysis using either a SmartCycler II System (Cepheid) or a Step One Plus real-time PCR platform supported with SDS 2.3 software (Life Technologies). The qualities of total RNA preparations were assessed by RT-qPCR targeting beta-Actin mRNA using primers (forward: 5`- TGACATCAAGGAGAAGCTCTGC and reverse: 5`- CCGCGGTGGCCATCT) and probe (5`FAM- ACGTGGCCCTGGACTTCGAGCA-BHQ-1) designed by M.Y. Deng of FADDL (unpublished protocol). Actin RT-qPCR was performed using AgPath-ID one-step RT-PCR reagents (Life Technologies) with cycling parameters: 48°C for 10 min, 95°C for 10 min, 45 cycles of: 95°C for 2 sec, and 60°C for 40 sec.

### Illumina Sequencing

Virus isolates tested positive by SV-A qRT-PCR were processed for Next Generation Sequencing (NGS) for complete genome sequencing based on the method described by Wang et al 2003 [20]. Briefly, total nucleic acid was extracted using the MagMax Viral RNA isolation kit (Life Technologies). RNA was quantified using a Qubit flourometer (Invitrogen). First strand synthesis of cDNA was performed with random primers and Superscript III reverse transcriptase (Invitrogen) for 50°C for 30 min, followed by incubation for 5 min at 65°C. Immediately afterwards, an additional 1ul of Superscript III was added to the reaction mix and incubated for 30 min at 50°C. Second strand synthesis was performed by addition of Sequenase enzyme (Affymetrix Santa Clara, CA); followed by a ramped incubation to 37°C for 16 min followed by 2 min at 94°C. Subsequently, the 37°C incubation was repeated with the addition of Sequenase, and the final product was amplified using TaqR mastermix (Clontech/Takara) using manufacturers’ recommended conditions. The resulting double stranded amplicon was processed for producing the sequencing library using a Nextera DNA sample preparation kit (Illumina) according to the manufacturers’ protocol. Sequencing was performed with a 600-cycle MiSeq sequencing kit (v3), and run on a MiSeq (v2) instrument.

## Results

### RT-qPCR Detection of SV-A

**Fig 1:**
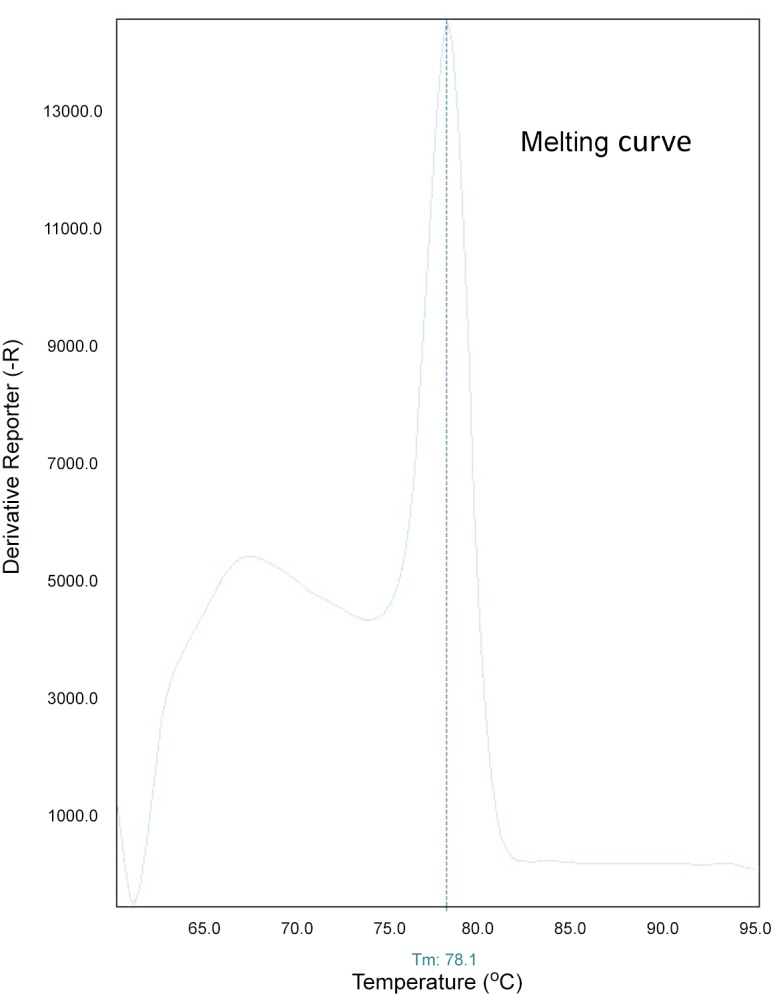
Melting curve for SV-A qPCR on the Step One Plus. Standard plasmid DNA containing SV-A gene fragment was used as the template for amplification in a SYBR green based qPCR reaction. RT-qPCR product had a melting temperature of ~78°C.

**Fig 2:**
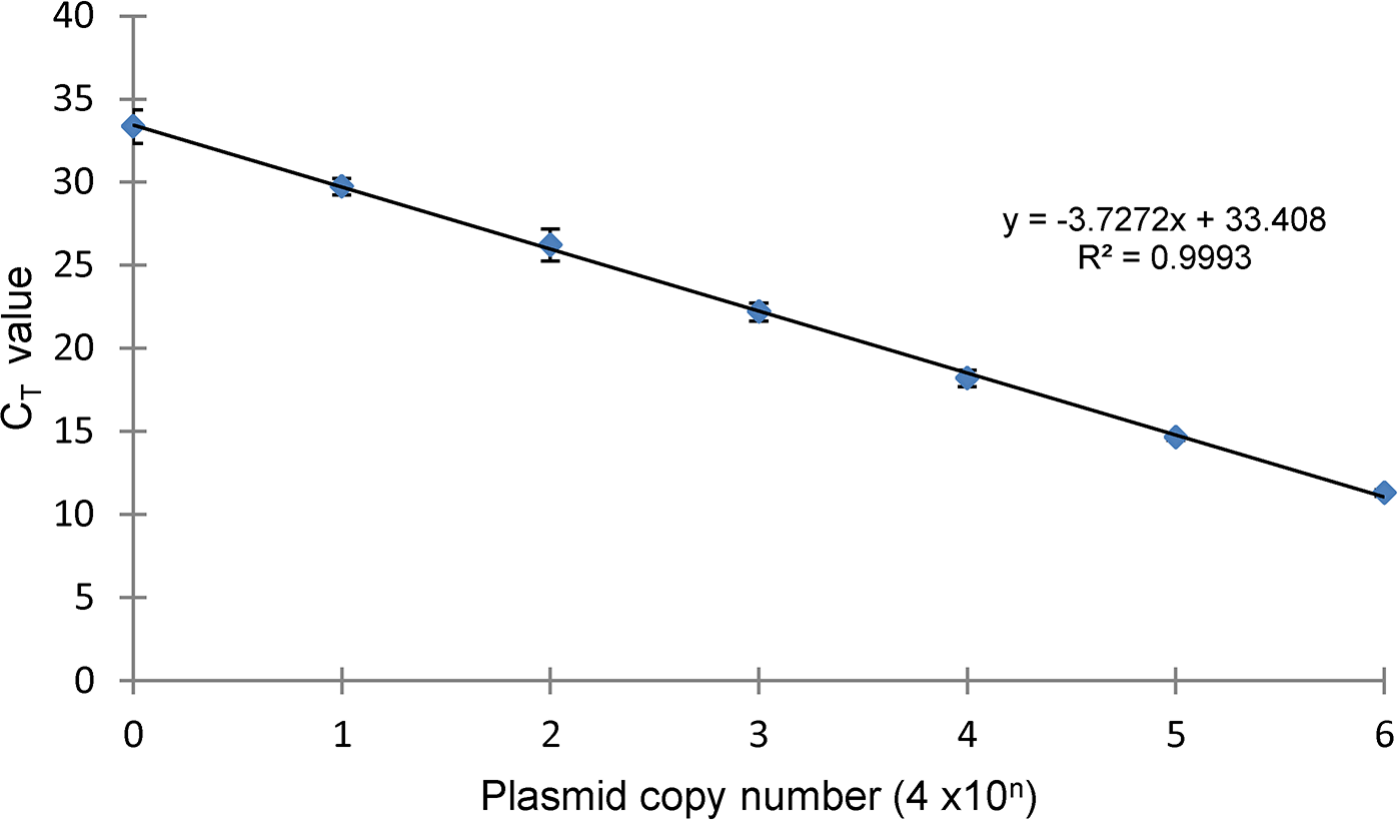
DNA doubling efficiency. Linear amplification of circular plasmid DNA was between copy numbers of 4 x 10^0^ and 4 x 10^6^, and generated a standard curve with calculated efficiency of 86% from y-slope (-3.72) with correlation coefficient (R^2^) of 0.9993.

**Fig 3:**
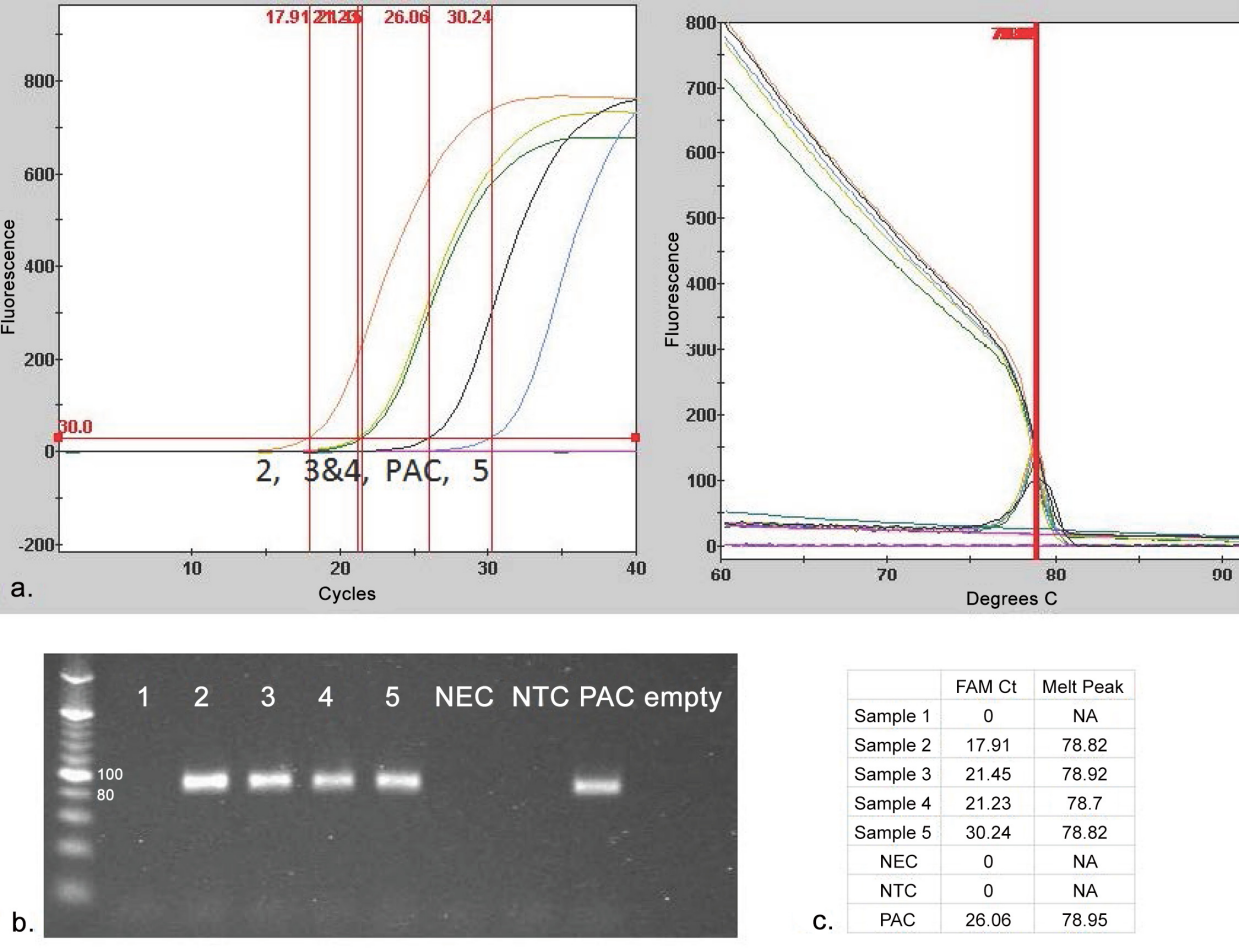
RT-qPCR data with amplicons visualized by DNA gel electrophoresis. Five diagnostic RNA samples with three controls were analyzed by RT-qPCR on the SmartCyclerII. (a) Ct and melt curves are displayed. (b) All products were subjected to high resolution gel electrophoresis to verify amplification of specific products of ~90bp. Control samples included a negative extraction (NEC), a negative template (NTC) and a positive amplification (PAC) control. (c) Ct values and melting temperature data is summarized.

Various SV-A sequences available in Genbank were aligned to identify a conserved primer set targeting an 87 bp sequence in the VP1 coding region suitable for RT-qPCR amplification (data not shown). Since sequence from different isolates displayed mismatches within the target 87 bp amplicon, DNA intercalating SYBR Green I dye was used for detection instead of a hybridizing labeled probe. A recombinant plasmid DNA constructed with a 983 bp SV-A sequence was used as the template to test the efficiency of qPCR primers. Amplification of a ~ 90 bp product was visualized on 2% agarose gel, and target sequence was confirmed (data not shown). A post-PCR dissociation curve analysis was included to confirm the specificity of amplification and yielded a sharp single peak with a melting temperature (Tm) of 78°C (Fig 1). A linear standard curve (mean Ct vs. log plasmid number) spanning six-orders of magnitude was obtained by amplification of 10-fold serial dilutions of plasmid DNA within the copy number range of 4 x 10^0^ to 4 x 10^6^ (Fig 2). Doubling efficiency of the amplified target DNA was estimated to be 86% from the y-slope (-3.72) with a regression R^2^ value greater than 0.99 (Fig 2). Diagnostic RNA samples with controls were analyzed by RT-qPCR, and the products were subsequently visualized by DNA gel electrophoresis to demonstrate correlation of amplification products to the presence or absence of a specific target. The RNA samples which displayed both positive Ct and Tm values within range of the positive amplification control generated ~90bp products when analyzed by gel electrophoresis (Fig 3).

### RT-qPCR Linearity and Reproducibility

**Fig 4:**
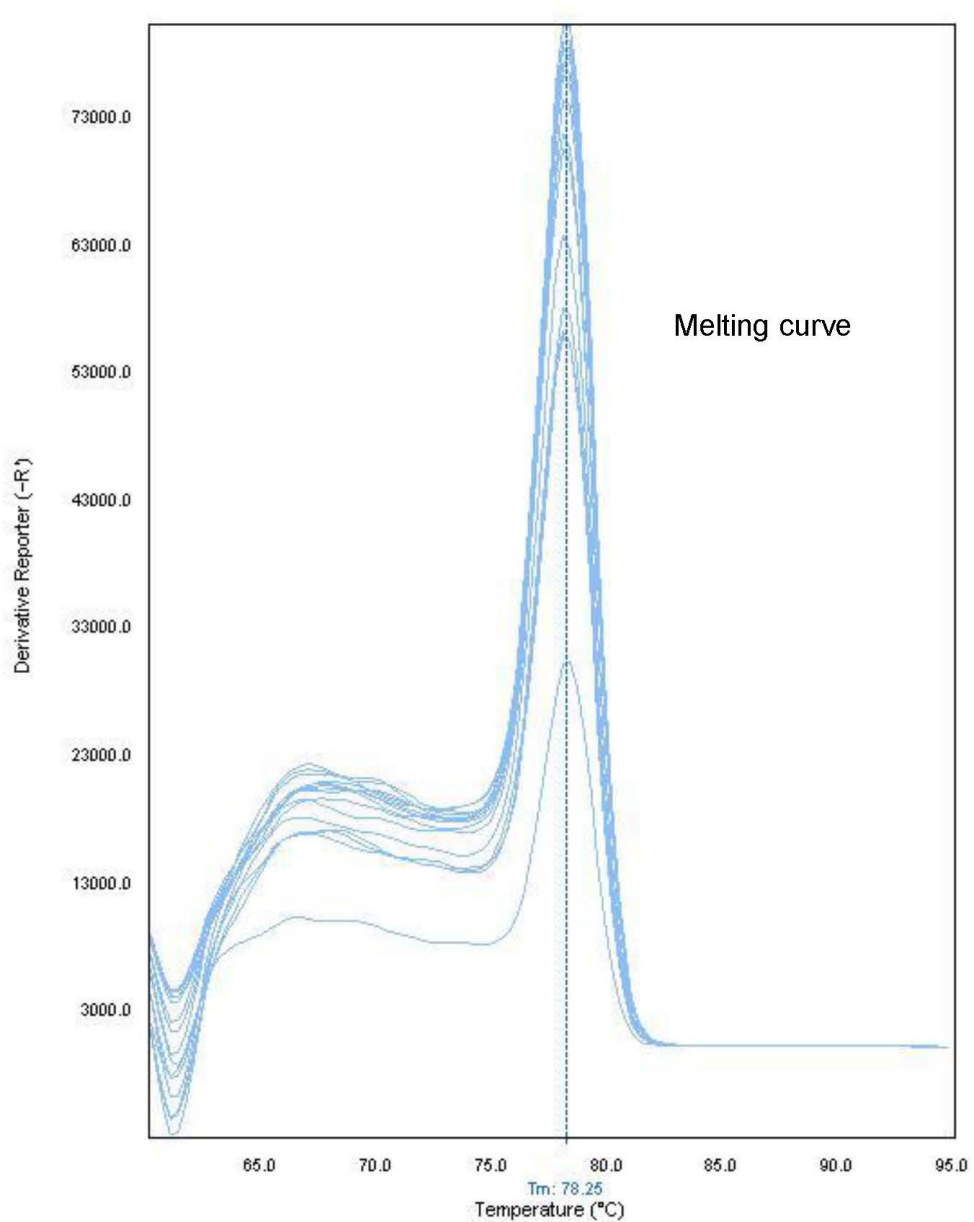
Composite overlay of RT-qPCR melting curves performed by the Step One Plus. Ten-fold serial dilutions of standard RNA control generated a unique melting point temperature (~Tm 78°C) for all concentrations of template.

**Fig 5:**
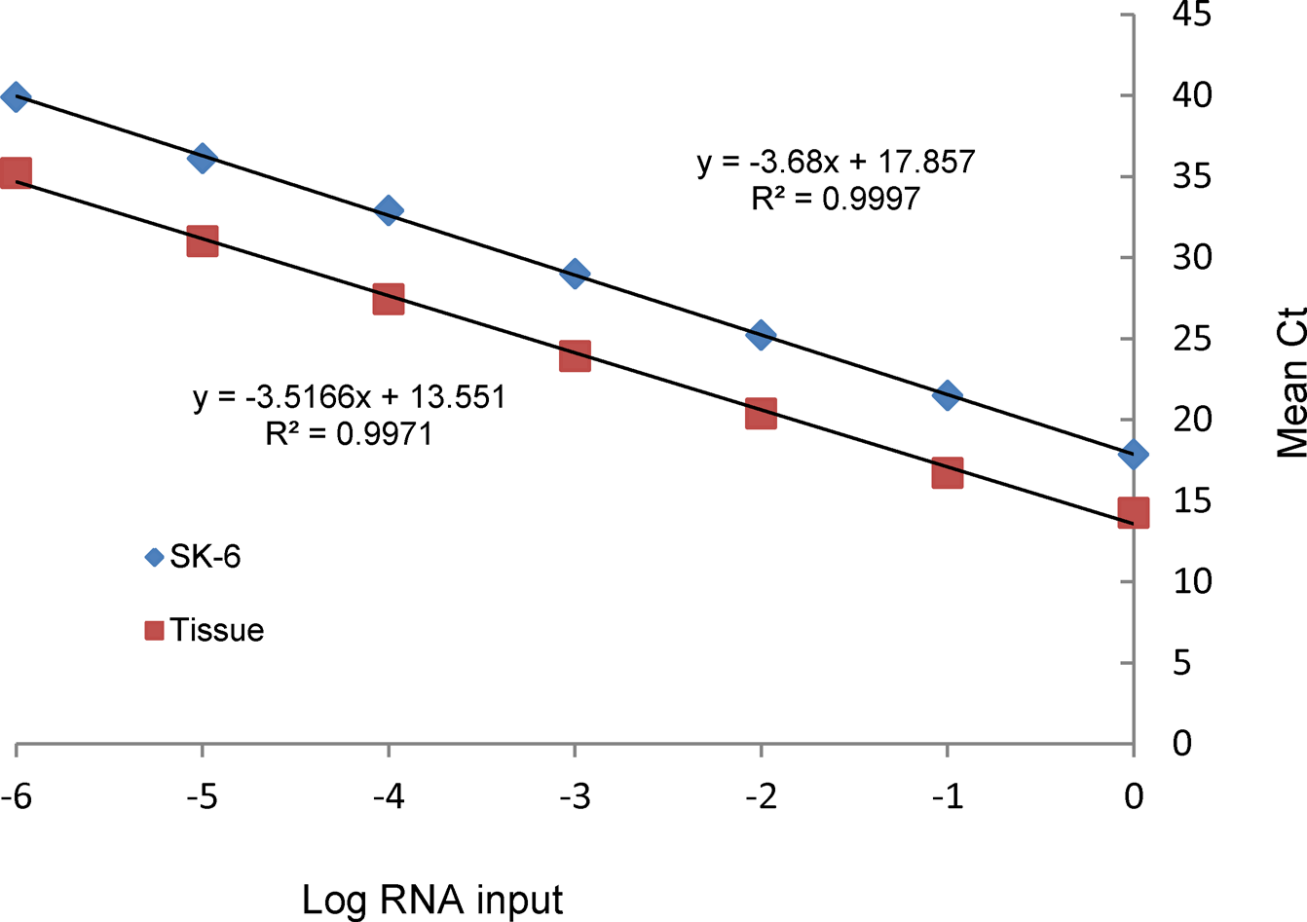
RT-qPCR linearity and sensitivity. Serial dilutions of standard control and SV-A positive tissue RNA produced linear standard amplification plot up to extinction with y-slopes of (-3.68 and -3.51, respectively) demonstrated >90% RT-qPCR efficiency with r^2^ > 0.99.

Three independent RNA extractions were performed from CPE-positive SK-6 cell cultures infected with SVV (2007 isolate). Ten-fold serial dilutions of total RNA preparations were subjected to RT-qPCR that yielded a defined melting curve with a peak at ~78°C (using the Step One Plus platform) for all dilutions which yielded a positive Ct value, suggesting consistency in melting point detection method over a wide range of target RNA concentrations (Fig 4). Standard curve plot of mean Ct value vs. log RNA input from three independent series of RNA dilutions showed linearity of RT-qPCR detection over six orders of magnitude (Fig 5). The slope value of -3.68 with R^2^ >0.99 represented a 90% efficiency in doubling of RT-qPCR. When compared to conventional RT-PCR, 983 bp products were observed from dilutions up to four orders of magnitude (data not shown). Consistency of the RT-qPCR assay was also examined using RNA preparations from diagnostic tissues collected in 2013 from a pig with vesicular lesions. Three independent series of 10-fold dilutions of RNA were subjected to RT-qPCR. The mean Ct value with negligible deviations from triplicate assays was plotted against logarithmic inputs of RNA template that gave a standard line with a slope of -3.51 (i.e., ~92% PCR efficiency) while R^2^ value was greater than 0.99 (Fig 5).

### RT-qPCR Specificity

To assess the selected RT-qPCR primers for cross amplification of sequences from non-target viral and host nucleic acids, a well characterized in-house reference panel of 29 extracted viral nucleic acids from clinical animal tissues or susceptible cell cultures was analyzed. Nucleic acid preparations representing positive amplification controls for 21 RNA and 8 DNA viruses were subjected to the SV-A RT-qPCR assay (Table 1), none of which yielded amplification or characteristic melting curve signals, suggesting that the primer set selected was highly specific for SV-A sequence detection (data not shown).

**Table 1:** Viruses tested by SV-A RT-qPCR assay.

*DNA viruses*	*RNA viruses*
African Swine Fever	Bovine Rhinovirus
Alcelaphine Herpes 1	Bovine Viral Diarrhea
Ovine Herpes 2	Classical Swine Fever
Swine Pox	Foot and Mouth Disease
Porcine Circovirus type 1 & 2	Pests des petits Ruminants
Pseudorabies	Porcine Reproductive and Respiratory Syndrome
Porcine Parvovirus	Porcine Enterovirus 1 & 8
	Porcine Epidemic Diarrhea
	Porcine Respiratory Coronavirus
	Porcine Teschovirus 1
	Rotavirus
	Swine Hepatitis E
	Swine Influenza
	Swine Vesicular Disease
	Transmissible Gastroenteritis
	Vesicular Exanthema of Swine
	Vesicular Stomatitis (New Jersey)
	Vesicular Stomatitis (Indiana 1, 2 & 3)

### Detection of SV-A in Swine Tissues

The RT-qPCR assay was used to test for the presence of SV-A RNA in 85 field tissue specimens collected over 10 years (2003–2013) from 50 swine with clinical features of vesicular diseases. All of these diagnostic specimens, listed in Table 2, were previously diagnosed negative for the presence of high-consequence porcine vesicular diseases including FMDV, VSV, VESV, and SVDV, but all possible endemic vesicular disease rule outs were not evaluated. A total of 67 (79% of total specimens analyzed) diagnostic specimens, representing 44 (i.e., 88% of animals tested) clinical animals, showed detectable amplification plots with Ct values ranging between 11 to 38 and Tm values of approximately 78°C, indicating detectable SV-A in those specimens (summarized in Table 2). A slight variation in Tm of products was observed and expected due to known variations in base composition of target amplicon sequences among different isolates and also due to differences of ramping and Tm collection rates of the thermal cyclers used. Thus, the products which contained a single melting peak with positive CT values produced Tm values within the range of 77.7 to 80.8°C, with a mean of 79.3°C. Melting temperature parameters fell within the range of 79.3°C ±1.6°C. SV-A RNA was found in a variety of different types of clinical specimens including vesicular fluid, epithelium, lymph node, swabs and epithelium from hoof lesion, etc., (Table 2). Of note, many of the vesicular diagnostic specimens were also found positive for the presence of one or more common viruses such as circovirus, parvovirus, enterovirus, pestivirus, etc., suggesting frequent co-infections of SV-A with other porcine viruses in field animals. Thirteen RT-qPCR positive samples were also tested by the conventional RT-PCR assay, confirming SV-A presence in all samples. Similarly, 18 RT-qPCR negative samples were confirmed for absence of SV-A particles by conventional RT-PCR assay and RNA integrity was confirmed present using the beta-actin RT-qPCR as previously described (data not shown).

**Table 2:** SV-A in swine vesicular diagnostic tissues.

Sample type	# Specimens	# RT-qPCR positive (CT<38 with corresponding Tm peak of 79.6 ±1.6°C)	
lymph node/spleen	29	24	
probang	7	6	
epithelium	17	12	
tonsil	6	4	
lung	3	1	
blood	1	1	
nasal swab	1	1	
kidney	1	0	
liver	1	0	
pooled vesicular sample (vesicular fluid, tissue and/or lesion swab)	19	18	
		
**Total # diagnostic specimens**	**85**	**67**	(79%)
**Total # clinical animals**	**50**	**44**	(88%)

**Fig 6:**
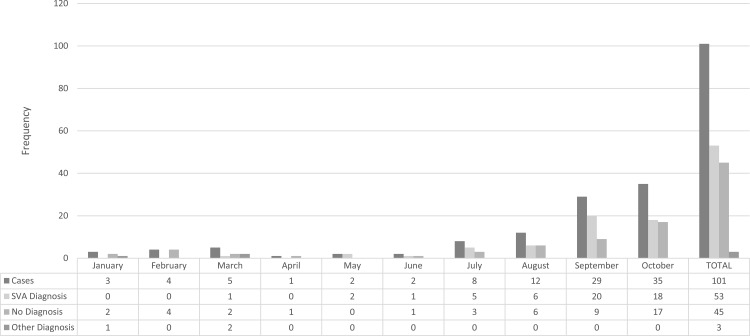
Summary of the 2015 swine vesicular case investigation results from FADDL, January to October 2015. During 2015 there was an increase in the submission of swine vesicular case investigations to FADDL. The SV-A qRT-PCR was regularly used starting in June 2015 on all investigation submissions. It resulted in the detection of 52 SV-A positive investigations out of the 101 investigations submitted during that time period. All high consequence diseases were also ruled out.

A control panel comprised of tissues from 35 swine known to be free of clinical vesicular disease were tested and found negative for the presence of SV-A RNA by RT-qPCR (Table 3). While none of the negative cohort tissue specimens yielded detectable SV-A RNA, 121 serum specimens collected from domestic and feral swine with or without clinical complications of vesicular lesions were also tested for SV-A particles and 18% from clinically diseased animals displayed very low amplification plots (i.e., high Ct values near 38) and produced expected positive melting curves, suggesting a weak but detectable presence of SV-A RNA in these animals (Table 3). Similar weak detection was also evidenced in 5.5% of serum from disease-free animals, of 47 feral and 35 domestic species, 6 (3 from each group) were found to contain SV-A RNA by RT-qPCR (Table 3). In 2013, 54 swine vesicular diagnostic specimens submitted for SV-A analysis were found to be positive by qRT-PCR. In 2014, only 14 specimens submitted for SV-A analysis were positive by qRT-PCR, and as of October 2015, 53 vesicular case investigations out 101 received by FADDL, have been determined positive by qRT-PCR (Fig 6). In total, 216 specimens submitted have been determined SV-A positive by qRT-PCR, and over 159 specimens have been confirmed SV-A qRT-PCR positive after virus isolation through November 2015 (Table 4).

**Table 3:** Summary of SV-A in various specimen types.

# Specimens	Source	Sample type	Vesicular sign	# Positive	% Positive
85	swine	tissue	clinical	67	79
35	swine	tissue	non clinical	0	
39	swine	serum	clinical	7	18
35	swine	serum	non clinical	3	9
47	feral swine	serum	non clinical	3	6
37	bovine	tissue	clinical	0	

**Table 4:** U.S. vesicular investigations diagnosed to be positive for SV-A via qRT-PCR by FADDL, 2013–2015.

Year	Positive investigations	Total # investigations	Positive specimens	Total # specimens
2013	11	17	54	81
2014	2	5	14	31
2015^a^	64	102	216	459

^a^ through November

### Sequence Analysis

**Fig 7:**
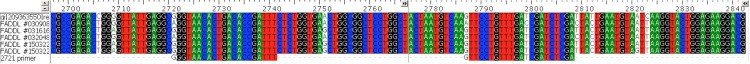
Sequence alignment of qRT-PCR viral isolates to SVV-001 and qRT-PCR primers. Five viral isolates were confirmed positive by qRT-PCR and the genome was sequenced. The amplification region was aligned with the qRT-PCR SV-A primers used in the assay, and to the reference sequence SVV-001: NC_011349.1 (NCBI).

Sequence data were obtained from the MiSeq instrument as raw, paired end reads in fastq format. Subsequent sequence assembly was produced through a reference guided assembly using BWAMEM from the Burrows-Wheeler alignment (BWA) software [21]. Assembled SV-A sequences were aligned using MUSCLE [22], and visualized with BioEdit (7.2.5) software [23] for comparison of the primer binding regions of the completed genomes (Fig 7).

### SV-A Not Detected in Bovine Tissues

The possible cross amplification of other vesicular etiological agents was examined by using vesicular diagnostic specimens from bovine, a species not considered susceptible to SV-A infection. A total of 37 archived bovine tissues were analyzed (including vesicular fluid, swabs and epithelium from hoof lesions, epithelium, nasal swab, oral probang sample, etc.), from 33 animals with signs of vesicular disease, and found to be negative for high consequence agents such as FMDV and VSV, although some of them were positively diagnosed for the presence of other viruses such as bluetongue virus (BTV), epizootic hemorrhagic disease virus (EHDV), and bovine popular stomatitis virus; strains found in the US domestic animal population (data not shown). These specimens were all found to be negative for SV-A RNA with this assay, indicating that the RT-qPCR primers selected in this study do not cross-react with common viruses responsible for look-a-like vesicular diseases in cattle.

## Discussion

Detection of SV-A antibodies in field animals with vesicular lesions has been attributed to possible association of the virus with SIVD syndrome [4, 5]. Differential diagnosis of SIVD from high consequence vesicular animal diseases, such as FMD, is crucial due to trade and movement restriction and other economic impacts associated with foreign animal diseases. Therefore, FADDL routinely investigates the presence of SV-A as the possible cause of vesicular lesions in swine while performing priority diagnosis from other devastating vesicular diseases including FMD by respective disease-specific RT-qPCR assays. The SV-A RT-qPCR test developed in this study complemented our laboratory needs for a fast, specific, sensitive and quantitative assay. Once demonstrated as sensitive, specific and reproducible in feasibility studies, we applied it to analyze swine vesicular diagnostic specimens that were previously found negative of the foreign animal disease agents FMD, VS, SVD and VES. Surprisingly, 88% of animals with vesicular lesions were positive for SV-A by RT-qPCR, confirming the previous observation that the virus appears to be associated with SIVD [7]. The SV-A conventional PCR was previously performed upon request only, and the SVV qRT-PCR assay was only performed regularly starting in June 2015 on all swine vesicular investigation submissions, due to the increased prevalence of the virus. The virus was detected in several types of tissue specimens suggesting that it is ubiquitously distributed in different organs of an infected animal. Some of the RNA templates demonstrated very low Ct values (<14) suggesting a high presence of SV-A particles in those specimens. Additionally, the virus could not be detected in any of the tissues from the 35 pigs that did not possess SIVD signature clinical signs.

Previously, the presence of SV-A neutralizing antibody was found in sera from pigs irrespective of the disease status [http://www.europic.org.uk/Europic2006/posters/Knowles.svv.01.pdf]. In this study, we were able to directly detect the low levels of SV-A RNA, presumably viral genome, in 27% of sera from swine with or without SIVD symptoms. This included 3 feral swine indicating that wildlife can serve as the possible natural reservoir of the virus. Recently, Yang et al 2012 [6] described the development of a SV-A indirect and cELISA assays which could be used for specific detection of SV-A serum antibodies. While farm animals are routinely monitored for appearance of vesicular lesions and precautionary laboratory diagnoses are done on suspect animals, feral swine are less frequently observed for vesicular disease. Pathogenicity of the virus remains unknown, and a cause for the observed high frequency of SV-A infection in animals presenting with vesicular disease is not yet understood. It is likely that environmental stressors or other infectious agents may be required in addition to SV-A for induction of SIVD. This assumption is supported by our frequent detection of other porcine viruses along with SV-A in clinical field animals and on an observed correlation from case histories indicating stressful conditions, such as movement, and the presence of SV-A in vesicular tissues presented in Table 2. An additional factor complicating the understanding of pathogenicity is the potential for sequence variation between strains of SV-A. Employing sensitive and specific detection methods for SV-A in rule-out diagnostic investigations of high consequence vesicular diseases in swine may not only help to resolve such disease investigations but ultimately may help to better understand the factors contributing to the presentation of SIVD.
